# Draft genomes of two Australian strains of the plant pathogen, *Phytophthora cinnamomi*

**DOI:** 10.12688/f1000research.12867.2

**Published:** 2018-02-28

**Authors:** Amy L. Longmuir, Peter L. Beech, Mark F. Richardson

**Affiliations:** 1 Centre for Cellular and Molecular Biology, School of Life and Environmental Sciences, Deakin University, Burwood, VIC, 3125, Australia; 2 Bioinformatics Core Research Group, School of Life and Environmental Sciences, Deakin University, Geelong, VIC, 3220, Australia; 3 Centre for Integrative Ecology, School of Life and Environmental Sciences, Deakin University, Geelong, VIC, 3220, Australia

**Keywords:** Phytophthora genome, plant pathogen, Phytophthora cinnamomi

## Abstract

**Background:** The oomycete plant pathogen, *Phytophthora cinnamomi*, is responsible for the destruction of thousands of species of native Australian plants, as well as several crops, such as avocado and macadamia, and has one of the widest host-plant ranges of the *Phytophthora* genus. The current reference genome of *P. cinnamomi *is based on an atypical strain and has large gaps in its assembly. To further studies of the pathogenicity of this species, especially in Australia, robust genome assemblies of more typical strains are required. Here we report the genome sequencing, draft assembly, and preliminary annotation of two geographically separated Australian strains of *P. cinnamomi*.

**Findings: ** Some 308 million raw reads were generated for the two strains, DU054 and WA94.26. Independent genome assembly produced final genome sequences of 62.8 Mb (in 14,268 scaffolds) and 68.1 Mb (in 10,084 scaffolds), which are comparable in size and contiguity to other *Phytophthora* genomes. Gene prediction yielded > 22,000 predicted protein-encoding genes within each genome, while BUSCO assessment showed 94.4% and 91.5% of the stramenopile single-copy orthologs to be present in the assembled genomes, respectively.

**Conclusions: **The assembled genomes of two geographically distant isolates of *Phytophthora cinnamomi *will provide a valuable resource for further comparative analyses and evolutionary studies of this destructive pathogen, and further annotation of the presented genomes may yield possible targets for novel pathogen control methods.

## Introduction

*Phytophthora cinnamomi* is a highly virulent plant pathogen that has a devastating impact on the Australian ecosystem, namely in the south-western areas of Western Australia and much of the south and east coasts of Victoria and New South Wales ^1^. In the south west ecoregion of Western Australia, alone, over 40% of the 5710 plant species present have been shown to be susceptible to *P. cinnamomi* ^2^. Significant genetic and phenotypic variation can occur within a signal clonal linage of *P. cinnamomi* ^3^ and susceptibility of a given host plant species has been shown to vary from site to site ^4^. Furthermore, despite the general lack of crossing during sexual reproduction, *P. cinnamomi* excels at adapting to new environments and developing virulence to new host species through asexual growth, making it a deadly and difficult-to-control pathogen. Unravelling how *P. cinnamomi* is able to adapt so quickly, and remain virulent, to a wide range of hosts in Australia, is an important research goal.

Currently, three *P. cinnamomi* strains have genome assemblies (MP94.48 and NZFS375, see 5 and Joint Genome Institute (JGI); NCBI Accession no. PRJNA68241). However, only the genome of *P. cinnamomi* var. *cinnamomi* (JGI; NCBI Accession no. PRJNA68241) has a publically available annotation, serving as the species reference genome. The assembly is based on the Rands isolate from Sumatra in 1922, which has been in culture for many decades and may not be representative of the current pathogenic strains present in Australia. Here we report and make available two Australian *P. cinnamomi* genomes, isolated from geographically very separate areas with different available host species. After analyses of genetic differences between these two *P. cinnamomi* genomes, it may be that key genes or gene families under high evolutionary pressure can be identified; this may aid further studies on more effective control of this pathogen.

## Sample collection and sequencing

Two isolates of *P. cinnamomi* were selected from areas of infection on either side of the Australian continent: one from the Brisbane Ranges in southeastern Australia (DU054, A2 mating type) ^6^ and the other from southwestern Western Australia (WA94.26, A2 mating type), both Deakin University culture collection. These isolates were selected to represent possible genetic diversity of *P. cinnamomi* in Australia arising from geographic isolation, and possible variation of selective pressures due to different host species. Isolates were maintained on V8 agar (V8A) [50 ml unclarified V8 ‘Original’ Juice (Campbells, Australia), 0.5 g CaCO _3_ and 7.5 g biological agar per 500 mL of distilled water] at 25°C in darkness, as per 6. Genomic DNA was isolated from hyphae using a DNeasy Plant Mini Kit (Qiagen), following the manufacturers’ protocol. Illumina TruSeq Nano library preparation (one per isolate) and sequencing on an Illumina HiSeq 2500 platform were performed by the Australian Genome Resources Facility (Walter and Eliza Hall Institute, Parkville, Australia) generating ~154 million paired-end (2 × 150 bp) raw reads per isolate. Raw reads are available in the NCBI Short Read Archive (SRA) under the Bioproject Accession: PRJNA413098.

## Genome assembly

Raw sequencing data for each isolate were first pre-processed using Trimmomatic v0.33 ^7^ with the following parameter values: *ILLUMINACLIP:TruSeq3-PE-2.fa:2:30:10:4 AVGQUAL:30 MINLEN:36*, to remove Illumina adapters and filter reads based on quality scores (Phred score). Only reads with average Phred > 30 were retained. To ensure only the desired *P. cinnamomi* genomes were assembled, a second round of pre-processing was conducted to remove potential contaminants. MetaPhlAn v2 ^8^, was run with default settings and identified the *Paenibacillus* genus as the only likely bacterial contaminate. Using BBMap v0.35 ( BBMap - Bushnell. B), we mapped the Trimmomatic-filtered reads to the closest species match ( *Paenibacillus* sp., JDR-2, GenBank accession: GCA_000023585.1, with 2.7% and 2.0% of DU054 and WA94.26 reads mapping, respectively; these *Paenibacillus* reads were subsequently removed. The remaining reads were then mapped using BBMap to the human genome (GRCh38; NCBI accession: GCA_000001405.15), with < 0.5% (~ 430,000 reads from DU054 and ~ 630,000 from WA94.26) being mapped and subsequently removed from the data set. Thus, the final set of reads (DU054, 149 million reads; WA94.26, 151 million reads) used for the assembly contained high-quality paired-end reads not belonging to either human or bacterial contaminants.

*De novo* contig assembly of the two genomes was conducted independently, using IDBA-UD v1.1.0 ^9^. IDBA-UD was run using the following parameter values: *--mink 20 --maxk 100 --step 20 --min_contig 500 --min_support 2 --min_count 3*. Briefly, these conducted a multiple K-mer assembly from k = 20 up to k = 100; only assembled contigs above 500 bp and those with a minimum depth coverage ≥ 3 were kept. As heterogeneous data can increase redundancy in genome assemblies (through heterozygous regions being assembled as separate contigs that results in highly fragmented assemblies ^10^), the IDBA-UD assembled contigs were run through the Redundans pipeline v0.12c ^10^ with the following parameter values: *-threads 4 -min_length 500*. Redundans uses paired-end mapping data to reduce assembled sequence redundancy and scaffold contigs into longer less fragmented sequences. The final assembled genome sequence of DU054 was 62.80 Mb in 14,269 scaffolds with an N50 of 9,951 bp; the longest scaffold was 1.54 Mb in length ( Table 1). For WA94.26, the final genome sequence was 68.07 Mb in length, in 10,085 scaffolds with the largest being 1.54 Mb and an N50 of 20,813 bp. GC content remained consistent, at ~ 53%, between both isolate genomes across both assemblies and before and after processing with Redundans. The quality, as measured by the above metrics, of the presented genomes is comparable to the previously available *P. cinnamomi* var. *cinnamomi* Rands isolate genome (JGI). The final genome assemblies are available under the NCBI Bioproject Accession: PRJNA413098.

**Table 1.** Summary of genomic features of assembled genomes comparing IDBA-UD output to scaffolded genome after Redundans processing and the *P. cinnamomi* Rands isolate genome.

	DU054		WA94.26	
	IDBA-UD	Redundans	IDBA-UD	Redundans
**Assemblysize (Mb)**	71.29	62.80	76.95	68.07
**No. scaffolds**	33,475	14,268	36,333	10,084
**N50 (bp)**	4,085	9,951	4,075	20,813
**No. predicted genes**	NA	23,414	NA	22,573

We used the BUSCO (benchmarking universal single-copy orthologs) pipeline v3.02 ^11^ in genome mode, with the default e-value cutoff of 0.01, to assess the completeness of the assembled genomes and compared the results to the previously available Rands isolate and the *P. cinnamomi* assemblies from Studholme *et al.* ^5^. Utilizing the set of 234 conserved stramenopile single-copy orthologs (hereafter BUSCOs), the analysis indicated 94.4% and 91.5% BUSCO completeness for the DU054 and WA94.26 genomes, respectively. For DU054, 221 complete BUSCOs (all single-copy with no duplicated BUSCOs) and 3 fragmented BUSCOs were identified, and 214 complete and 2 fragmented BUSCOs in WA94.26 ( Table 2). Overall, we find a higher level of BUSCO completeness compared with the Rands isolate, and comparable (albeit it slightly lower) completeness compared to the two *P. cinnamomi* assemblies from Studholme *et al.* ^5^ ( Table 2). This suggests our two Australian isolate assemblies are as complete references as those currently available.

**Table 2.** Summary of BUSCO assessment.

	DU054	WA94.26	*P. cinnamomi* var. *cinnamomi*	MP94.48 ^5^	NZFS375 ^5^
**Total** BUSCOs	234	234	234	234	234
**Complete and single copy** BUSCOs	221 (94.4%)	214 (91.5%)	202 (86.3%)	228 (97.4%)	228 (97.4%)
**Complete and duplicate** BUSCOs	0 (0%)	0 (0%)	4 (1.7%)	0 (0%)	0 (0%)
**Fragmented** BUSCOs	3 (1.3%)	2 (0.9%)	7 (3.0%)	2 (0.9%)	2 (0.9%)
**Missing** BUSCOs	10 (4.3%)	18 (7.6%)	21 (9.0%)	4 (1.7%)	4 (1.7%)

## Preliminary genome annotation

We conducted a preliminary protein-coding sequence prediction using GeneMark-ES v4.32 ^12^, which utilises a self-training algorithm to identify exon, intron and intergenic regions as well as initiation and termination sites. GeneMark-ES was run using the default settings and a database of predicted gene models (i.e., predicted polypeptides) was constructed for DU054 and WA94.26 genomes (available in the associated data repository ^13^). An initial 23,414 gene models were identified in DU054 and 22,573 in WA94.26. Of these, 14,735 pairs of predicted gene models appear to be orthologous between the two genomes (reciprocal best-hit Blastp, e value ≤ 1e-5). As a preliminary verification of these gene model builds, we identified orthologous counterparts to eight available *Phytophthora* genomes with annotations [ *P. infestans* ^14^, *P. kernoviae* ^15^, *P. lateralis* ^16^, *P. nicotianae* ^17^, *P. parasitica* (P1569_v1; Broad Institute), *P. ramorum* ^18^, *P. sojae* ^18^ and *P. cinnamomi* var. *cinnamomi*]. Accordingly, we used OrthoFinder v1.1.10 ^19^ with default parameter values, except we used DIAMOND ^20^ as the alignment program with the *diamond_more_sensitive* flag. OrthoFinder first identifies ‘orthogroups’ (an extension of orthologues to include groups of genes descended from a single gene in the last common ancestor of a group of species ^19^) and then orthologues between each pair of species in the comparison. OrthoFinder assigned 88.5% (170,769) of the genes found in all the species to 19,089 orthogroups, and of these 50% of all the genes were contained in orthogroups, which had 10 or more genes within them. We found 2,931 orthogroups that contained genes for each of the species, and of these 1,309 orthogroups consisted entirely of single-copy genes; see associated data repository ^13^. Using these single-copy orthogroups, gene trees were first constructed, then the species tree was inferred using the distance-based method implemented by fastme ^21^. The resultant species tree (see associated data repository ^13^) exhibits strong congruence to the *Phytophthora* phylogeny recently published by 22, providing more evidence that the genome assembly and preliminary annotation conducted here is valuable.

## Conclusions

In summary, we present the genome assembly of two geographically separated isolates of *Phytophthora cinnamomi* from Australia. These high-quality genome assemblies may act as a valuable resource for comparative genomics and particularly for the further identification and analysis of protein-encoding genes expressed during plant infection, such as members of the avirulence gene families ^23^. These gene families are of specific interest in the development of novel and effective pathogen control mechanisms.

## Data availability

The data referenced by this article are under copyright with the following copyright statement: Copyright: © 2018 Longmuir AL et al.

Data associated with the article are available under the terms of the Creative Commons Zero "No rights reserved" data waiver (CC0 1.0 Public domain dedication). 

Raw reads are available in the NCBI SRA under the Bioproject Accession: PRJNA413098.

The final assemblies are available at DDBJ/EMBL/GenBank under the accessions, PDCY00000000 and PDCZ00000000 and under the Bioproject Accession: PRJNA413098.

Supporting data, including preliminary gene prediction, OrthoFinder analysis and BUSCO assessment results can be found in the associated data repository: doi, 10.4225/16/59d15a6917a5e ^20^. Data are available under the terms of the Creative Commons Attribution 4.0 International (CC BY 4.0).
