# Supplementary material for: Draft genomes of two Australian strains of the plant pathogen, Phytophthora cinnamomi
Source: F1000Res. 2018 Feb 28;6:1972. Originally published 2017 Nov 8. [Version 2] doi: 10.12688/f1000research.12867.2 (PMC5698912; doi:10.12688/f1000research.12867.2)
Supplement: Peer review history

# Peer review: Referee response for version 2

**DOI:** 10.5256/f1000research.14404.r31293

**Published:** 2018-03-08

**Authors:** David J. Studholme

**Affiliations:** 1 Biosciences, University of Exeter, Exeter, UK

The authors have satisfactorily dealt with all the issues raised in my previous report.

I have read this submission. I believe that I have an appropriate level of expertise to confirm that it is of an acceptable scientific standard.

# Peer review: Referee response for version 1

**DOI:** 10.5256/f1000research.13945.r28065

**Published:** 2017-11-28

**Authors:** David J. Studholme

**Affiliations:** 1 Biosciences, University of Exeter, Exeter, UK

This manuscript announces the availability of genomic sequence data from Phytophthora cinnammomi strains DU054 and WA94.26. This is a useful resource for researchers interested in this important pathogen. The authors have deposited and made available the raw sequence data in SRA and their assemblies in GenBank, which is commendable. The genome annotation and protein sequences do not appear to be deposited in GenBank, however. This does not preclude publication, but when discussing predicted genes in the manuscript, the authors should be up-front about this or provide full details of the annotations in supplementary data or deposit them in some public repository.

 One oversight that the authors should be aware of is the previous publication of two genome sequences of this species, one of which (MP94-48) is from Australia. See Studholme *et al*. (2015) ^1^. So, the authors' assertion (in their Conclusions section) that this is the first genome assembly from an Australian strain should be revised. The authors should also include those two assemblies in their comparisons of assembly quality metrics. And also please revise the several other mentions of previously sequenced genome throughout the text in the light of the additional two previously sequenced genomes. Also, it would be interesting to assess how similar or different all these four available P.c. genome sequences are to each other, e.g. by calculating pairwise ANIs.

 Some specific points that should be addressed around the methodology:

1. Why were reads mapped against the human genome? Why should contamination from human DNA be more prevalent or likely than from other organisms?
2. The authors make good efforts to remove contaminating Paenibacillus sequence reads. Interestingly, we also observed contamination of Phytophthora genomic DNA with this bacterial genus. However, the authors go on to claim that the data contained "highly quality reads not belonging to ... bacterial contaminants". Their approach does not remove non-Paenibacillus bacterial contaminants.
3. Please cite a reference to support the claim that "heterogeneous data can increase redundancy in genome assemblies". It is not entirely clear what this statement means, precisely, and in any case it is not self-evident and needs to be supported by peer-reviewed publication.
4. The use of BUSCO version 1.22 is questionable, given that versions 2 and 3 are now available. Furthermore, rather than using the general Eukaryote set of BUSCOs, the authors should use the Stramenopile set.
5. The completeness of the genome assemblies is rather poor (only < 65% of expected genes are present intact in a single copy). It would be useful to compare/benchmark this against other available Phytophthora genome sequences. For example, our recent sequencing of P. ramorum genomes, we found around 81- 85% of Stramenopile BUSCOs were intact and single-copy in each genome (See PubMed ID 28243575).
6. Towards the end of page 4, the authors claim that the "preliminary annotation ... is valuable". I agree and would go further to say that not just the annotation but the genome sequencing per se is valuable. I would also suggest including a brief explanation of how/why the presented data is valuable. 
7. The authors say that their annotation is valuable, but the annotation has not apparently been deposited in a public repository. Therefore, please either make this valuable resource available, or remove the claim that it is valuable.

Some very minor points:

1. In the Introduction, it was not obvious to me what is meant by a "Botanical Province". Please consider explaining this term.
2. Please add an apostrophe after "manufacturers".
3. At several places in the text, the authors write "parameters" when they really mean "parameter values" or "options" or "switches". Please check and revise.
4. Please write "high-quality" not "highly quality".
5. On page 3, the authors say that no gene expression data are available for this species. This is untrue, since EST data (i.e. expressed sequence tags) are available. Furthermore, in the SRA, there are several RNAseq datasets available: 
Illumina HiSeq 2000 sequencing; RNAseq analysis of germinating cysts of Phytophthora cinnamomi 1 ILLUMINA (Illumina HiSeq 2000) run: 46,420 spots, 4.2M bases, 3.5Mb downloads Accession: ERX709652 Select item 14623972.
Illumina HiSeq 2000 paired end sequencing 1 ILLUMINA (Illumina HiSeq 2000) run: 9.9M spots, 1.8G bases, 1.1Gb downloads Accession: ERX943317 Select item 1426113.
Phytophthora cinnamomi library 1 ILLUMINA (Illumina HiSeq 2000) run: 88.1M spots, 17.6G bases, 10.3Gb downloads Accession: SRX124562 Select item 1426104.
Phytophthora cinnamomi library 1 ILLUMINA (Illumina HiSeq 2000) run: 30,453 spots, 6.1M bases, 2.6Mb downloads Accession: SRX124561 Select item 1426095.
Phytophthora cinnamomi library1 ILLUMINA (Illumina HiSeq 2000) run: 50.6M spots, 10.1G bases, 5.9Gb downloads Accession: SRX124560 Select item 1426086.
Phytophthora cinnamomi library 2 ILLUMINA (Illumina HiSeq 2000) runs: 38.5M spots, 7.7G bases, 4.5Gb downloads Accession:  SRX124559
6. ​​When quoting N50 values, please include the units. For example, the N50 for DU054 was 9,951 bp or nt.
7. The authors refer to (on page 4) "more complete annotations" of several species. Among these examples is P. lateralis and a citation of our paper (PubMed 23678994) about the sequencing of this species' genome; however, I would not agree that its annotation is "more complete".
8. On page 3, second paragraph, the authors write "the available genome". It is not the "genome" that is available; rather it is the "genome sequence".

Once the authors have addressed all these issues, I would be very pleased to see this indexed.

I have read this submission. I believe that I have an appropriate level of expertise to confirm that it is of an acceptable scientific standard, however I have significant reservations, as outlined above.

## References

[1] StudholmeDJMcDougalRLSamblesCHansenEHardyGGrantMGanleyRJWilliamsNM: Genome sequences of six Phytophthora species associated with forests in New Zealand. *Genom Data*.2016;7: 10.1016/j.gdata.2015.11.015 54-6 10.1016/j.gdata.2015.11.015 26981359PMC4778589

# Response

**Published:** 2018-02-28

**Authors:** Mark Richardson

**Affiliations:** Deakin University, Australia

Thank you very much for providing a thorough review and pointing out several oversights we made. We have endeavoured to rectify these as you will see from our responses below. Importantly, we have included the preliminary gene prediction results in the associated public repository with the current supplementary materials. We have also revised the manuscript to include the additional genomes from Studholme *et al.* 2015 and included them in a comparative BUSCO completeness assessment. We feel a more comprehensive comparative analysis (including ANIs) is beyond the scope of this research note, but this will be part of a future paper.

For clarity, the below responses are separated into Major and Minor subheadings and numbered as per points in the review in order to avoid duplication of text.

Major:

1. Contamination for human DNA should not be more prevalent than any other. As this was one of the first times we cultured this species we carried out this pre-filtering to ascertain whether or not we had any inadvertent contamination. The results show this was not the case.2. We find it interesting that the reviewer has also detected Paenibacillus contamination during their work.

2. While removing contamination through mapping to the Paenibacillus genome alone would not warrant our statement, this is not what we did. We used MetaPHlAn to first screen our raw reads to identify which, if any, bacterial species might be present. Only Paenibacillus could be detected. Thereby, once removed, we are confident that no other bacterial contamination exists. If others had been identified with MetaPHlA then they could be removed in the same way. 

3. We have added a citation to this extent and clarified what we mean in the text. 

4. We have repeated this analysis with version 3.02 and used the suggested ortholog set. 

5. With respect, we feel that the reviewer’s statement that the completeness was poor is unfounded, especially if we consider that they are not making a ‘like for like’ comparison by comparing results from the eukaryotic set to those from the stramenopile set. Nevertheless, the updated BUSCO analysis using the stramenopile set reveals the genome assemblies presented here have BUSCO completeness of ~91 to 94 %, which falls within the range for the previous *P. cinnamomi* assembles (86 -97% completeness, see Table 2 for full comparison). 

6. Thank you for this suggestion, we have done so. 

7. This is a very valid point. We have now included the preliminary gene predictions with the supplementary data. 

Minor:

1. We have changed this to the more commonly understood ‘ecoregion’
2. Done
3. Done
4. Done
5. We have removed this statement.
6. Done
7. We have removed this statement.
8. Addressed

# Peer review: Referee response for version 1

**DOI:** 10.5256/f1000research.13945.r27740

**Published:** 2017-11-20

**Authors:** Erik Andreasson, Laura Grenville Briggs

**Affiliations:** 1 Department of Plant Protection Biology, Swedish University of Agricultural Sciences, Alnarp, Sweden

This data adds information about this important organism in the standard format to report a draft genome these days so it looks fine. They used hiseq and sequence coverage (BUSCO) looks appropriate and expected although there are relatively large differences between the two isolates (i.e .different final genome sizes and busco completeness scores). One suggestion is to add information on how many libraries they sequenced, and if it was only paired end and not also mate paired.

We have read this submission. We believe that we have an appropriate level of expertise to confirm that it is of an acceptable scientific standard.

# Response

**Published:** 2018-02-28

**Authors:** Mark Richardson

**Affiliations:** Deakin University, Australia

Thank you for the positive review, we have added the additional information you have requested.

# Peer review: Referee response for version 1

**DOI:** 10.5256/f1000research.13945.r27739

**Published:** 2017-11-17

**Authors:** Nicolás Daniel Ayub

**Affiliations:** 1 National Scientific and Technical Research Council, Buenos Aires, Argentina

The work was carried out professionally and resulted in good draft genomes of two pathogen strains belonging to Phytophthora genus. In my opinion, this article is an important contribution to future studies about the molecular mechanism involved in *Phytophthora*-plant interaction. Particularly, in the first steps of pathogen adhesion, where the virulence factors related to this are little known.

I have read this submission. I believe that I have an appropriate level of expertise to confirm that it is of an acceptable scientific standard.

# Response

**Published:** 2018-02-28

**Authors:** Mark Richardson

**Affiliations:** Deakin University, Australia

Thank you for the positive review.
